# Changes of Physicochemical Indicators and Volatile Compounds in Grains and Liquors During the Sauce-Aroma *Baijiu* Brewing Process

**DOI:** 10.3390/foods15122211

**Published:** 2026-06-19

**Authors:** Shenglan Xu, Jun Xu, Qingshan Wu, Huimin Zhou, Die Lu, Lili Jia, Fusheng Chen

**Affiliations:** 1 School of Life Sciences, Guizhou Normal University, Guiyang 550025, China; 18334135539@163.com (S.X.); wqs288@126.com (Q.W.); 19185436624@163.com (D.L.); 2 Sichuan Liquor Group Co., Ltd., Chengdu 610041, China; 15997637283@163.com; 3 Hubei International Scientific and Technological Cooperation Base of Traditional Fermented Foods, Huazhong Agricultural University, Wuhan 430070, China; 18872971570@163.com; 4 College of Food Science and Technology, Huazhong Agricultural University, Wuhan 430070, China

**Keywords:** sauce-aroma *Baijiu*, physicochemical indicators, volatile compounds, HS-SPME-GC-MS, correlation

## Abstract

Sauce-aroma *Baijiu* is produced through a one-year cyclic process involving multiple fermentations and distillations. However, the dynamic changes and correlations among fermented grains (FG), distilled fermented grains (DG), heart liquor (HL) and tail liquor (TL) remain unclear. In this study, the physicochemical indicators and volatile compounds (VCs) from the 3rd to 6th distillation rounds were systematically analyzed. Across successive rounds, FG and DG exhibited similar trends in key physicochemical indicators, as did HL and TL. Headspace solid-phase microextraction coupled with gas chromatography–mass spectrometry (HS-SPME/GC-MS) identified 76, 73, 80 and 93 VCs in FG, DG, HL and TL, respectively. Multivariate statistical analyses revealed significant inter-round differences in volatile profiles, and further indicated that total acids and water contents in FG were positively correlated with the majority of VCs in liquor. These results clarify the dynamic change of physicochemical and flavor components during *Baijiu* production and provide a basis for quality evaluation.

## 1. Introduction

Chinese *Baijiu*, alongside brandy, whisky, rum, vodka, and gin, is recognized as one of the six most famous distilled spirits in the world [1]. It is produced primarily from cereal materials such as sorghum, rice and wheat, and is brewed via solid-state fermentation, distillation, aging and blending, with a production and consumption history over thousands of years [2,3,4]. Based on the flavor characteristics, Chinese *Baijiu* is classified into twelve aroma types: sauce-aroma, strong-aroma, light-aroma, rice-aroma, mixed-aroma, *Dong*-aroma, *Feng*-aroma, *Te*-aroma, sesame-aroma, *Laobaigan*-aroma, *Fuyu*-aroma and *Chi*-aroma [5]. Among these liquors, sauce-aroma *Baijiu* has gained wide consumer preference due to its distinctive flavor profile, which is dominated by a prominent “sauce” aroma, complemented by elegant and delicate notes, a mellow and full-bodied taste, and a prolonged aftertaste [5,6]. These flavor compounds, such as ethyl acetate, butanol and ethyl caprylate, exhibit significant positive correlations with enhanced ethanol clearance and better post-drink comfort [7]. Consequently, sauce-aroma *Baijiu* has commanded strong consumer demand, as reflected in its 2024 output of approximately 650,000 kiloliters and revenue of 240 billion yuan, underscoring its considerable importance in the overall liquor market [8].

**Figure 1:**
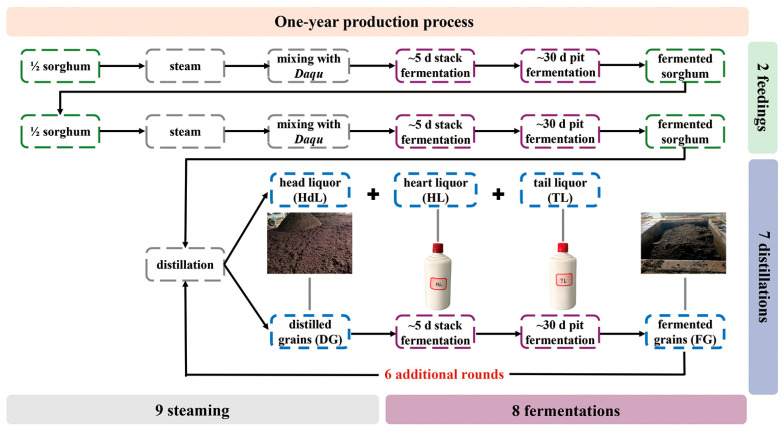
One-year production process of sauce-aroma *Baijiu*. Dashed boxes indicate the main steps of the brewing cycle. The process comprises two feedings (green), nine steaming (grey), eight fermentations (purple), and seven liquor extractions (blue). In the final cycle, the process terminates after steaming and liquor extraction, without further fermentation of distilled grains. The entire production process spans approximately one year.

The brewing process of sauce-aroma *Baijiu* is highly complex [4,9], which is typically made by a special type of glutinous sorghum with a hard and thick hull and amylopectin content exceeding 90%. As shown in Figure 1, the brewing process of sauce-aroma *Baijiu* generally begins at the Chinese *Chongyang* Festival (Senior’s Day), which falls on the ninth day of the ninth lunar month. At the beginning, half of the sorghum (raw material) is steamed and, after cooling to approximately 30 °C, mixed with the fermentation starter *Daqu*. The mixture is then stacked on the ground in a conical shape for about 5 days until the inner temperature rises to over 50 °C. This open process, known as stack fermentation, facilitates the recruitment of microorganisms from the surrounding environment [10]. After the stack fermentation, the material is transferred to a stone-lined underground pit (*Jiao*) for anaerobic fermentation. After approximately 30 days fermentation, the fermented material will be taken out and combined with the remaining half of the raw sorghum. The mixture is then again steamed, cooled and mixed with *Daqu*, subjected to another round of stack fermentation, and subsequently returned to the pit for continued anaerobic fermentation. The brewing process then enters a cyclic stage, with each fermentation cycle lasting about 35 days. At the end of each cycle, the fermented materials are taken out and distilled to obtain liquor fractions. The solid residues remaining after distillation are mixed with *Daqu*, undergo a short period of stack fermentation, and are then returned to the pit for the next round of anaerobic fermentation. This fermentation and distillation process is repeated for other 6 rounds, and after the final distillation, the solid residues are not reused for further fermentation [11]. Consequently, the entire brewing process of sauce-aroma *Baijiu* extends about “1” year, consisting of “2” material feedings, “9” steaming, “8” fermentations and “7” distillations to extract liquors, which is commonly referred to as the “12987” brewing technology of sauce-aroma *Baijiu* [4].

During the process of sauce-aroma *Baijiu*, the fermented materials directly retrieved from the pit after each cycle of fermentation is *Jiupei* and also named as fermented grains (FG). The FG is distilled to obtain the liquid fraction, namely raw liquor, while the remaining solid residue, termed *Jiuzao* and also known as distilled grains (DG), is reused in the next fermentation cycle. According to the sequence of outflow, the raw liquor can be divided into head liquor (HdL), heart liquor (HL) and tail liquor (TL). HdL and TL are liquid by-products of sauce-aroma *Baijiu*. HdL, the beginning distilled fraction of raw liquor, is characterized by a low yield, pungent and bitter taste, liquid turbidity, but high concentration of low-boiling-point VCs [12], and is mainly used for blending *Baijiu* final product. In contrast, the final fraction TL, has a high yield but low alcohol content, and is generally applied in a *Pojiao* process [10], in which TL is splashed onto the pit walls and bottom to inhibit harmful microorganisms and regulate moisture during the anaerobic fermentation. In addition, TL can also be utilized as a raw material for vinegar and other fermented products [13]. HL, the fraction collected after HdL, is the main part of raw liquor with a moderate alcohol concentration and relatively high yield. After being aged for at least three years, transformations such as esterification, hydrolysis, oxidation, Maillard reaction and volatilization occur and contribute to the development of desirable aroma [14,15], making the aged HL qualified to serves as the base liquor for producing the *Baijiu* final product.

Researches have proven that the composition and quality of HL differ substantially across different rounds. For instance, HL from the 1st and 2nd rounds possess a strong grain flavor, while those from the 3rd to 5th rounds are enriched in characteristic sauce-aroma VCs. In contrast, HL from the 6th and 7th rounds contain distinct roasted and baked flavors [2]. In the complex brewing system of sauce-aroma *Baijiu*, the presence and succession of microbial communities are the core driving forces for shaping its unique flavor profile. For instance, *Lactobacillus* spp. boosts the production of ethyl acetate and ethyl lactate, thereby enhancing the fruity aroma and establishing a balanced acid-ester profile in sauce-aroma *Baijiu* [16]. *Leuconostoc* spp. and *Bradyrhizobium* spp., both of which correlate positively with tetramethyl-pyrazine, an important flavor compound for sauce-aroma *Baijiu* [17]. On the other hand, throughout the entire fermentation process, the succession of the microbial community is tightly correlated with FG [18]. The microbial community in the FG is influenced by water contents, acidity, levels of reducing sugars and starch, as well as oxygen availability, leading to variations in both microbial population size and species composition that may ultimately affect the formation of flavor compounds in *Baijiu* [19,20]. Sauce-aroma *Baijiu* features a unique and highly complex multi-round fermentation and distillation process. However, existing studies have predominantly concentrated on single fermentation rounds or individual production stages [21,22], while the systematic characterization of the dynamic changes in physicochemical properties and volatile compounds at different production stages during multi-round fermentation, as well as the correlations among them, remain poorly understood.

This study aimed to elucidate the correlations between physicochemical factors and volatile compounds across different production stages during multi-round fermentation of sauce-aroma *Baijiu*, and to provide insights into the VC compositional differences of raw liquors across distillation rounds. To this end, the physicochemical indicators and VCs profiles in FG, DG, HL and TL from the 3rd to 6th rounds were systematically investigated and compared.

## 2. Materials and Methods

### 2.1. Experimental Materials

Samples of FG, DG, HL and TL were collected during the 3rd to 6th distillation rounds from a sauce-aroma *Baijiu* production company located in *Maotai* Town, *Guizhou* Province, China, a widely recognized representative production region for sauce-aroma *Baijiu*. Samples were labeled according to the material type and distillation round (e.g., FG3 represents FG collected from the 3rd distillation round). Three independent fermentation pits that fermented for one month from the same workshop were analyzed, and sampling was conducted in 2023. HL and TL were stored in opaque glass bottles, whereas FG and DG were collected in sterile bags. All samples were stored at 4 °C prior to analysis. For subsequent analyses, grain samples were thoroughly mashed and mixed, while liquor samples were homogenized before processing.

### 2.2. Determination of Physicochemical Indicators

#### 2.2.1. Water Contents

Water content was determined using the drying method. A pre-dried beaker of constant weight was filled with an appropriate amount of FG or DG and weighed, then dried at 100 °C to a constant weight. The water content was calculated according to Formula (1):

(1) $$\mathrm{Water}\;\mathrm{contents}\;(\%)=\frac{m_{1}\;-\;m_{2}}{m_{1}\;-\;m_{0}}\;\times \;100$$

where *m*_0_ is the weight of the weighing bottle (g); *m*_1_ is the weight of the bottle plus sample before drying (g); *m*_2_ is the weight after drying (g).

#### 2.2.2. Reducing Sugar Contents

Fresh FG and DG samples equivalent to 100 g dry weight were immersed in 1 L of distilled water for 30 min with stirring every 10 min. The mixture was filtered, and the filtrate volume was adjusted to 1 L with distilled water, yielding a solution equivalent to 0.1 g/mL FG or DG, and this solution was used for subsequent analyses.

The Fehling’s method was applied to detect the reducing sugar contents. Briefly, 5 mL each of Fehling’s reagent solution A and B (Shanghai Yaji Biological Technology Co., Ltd., Shanghai, China) was titrated with 1 mg/mL glucose standard solution until the blue color disappeared, and the consumed volume was recorded. For sample analysis, 2 mL of the filtrate, which is equivalent to 0.2 g of FG or DG, was titrated under same conditions as the standard solution. Reducing sugar contents was calculated by Formula (2):

(2) $$\mathrm{Reducing}\;\mathrm{sugars}\;(g/100\;g)=\frac{c\;\times \;(V_{0}-V_{1})\;\times \;100}{0.2}\;\times \;10^{-3}$$

where *c* is the glucose concentration (mg/mL); *V*_0_ is the volume of glucose solution consumed in blank titration (mL); and *V*_1_ is the volume consumed for sample titration (mL).

#### 2.2.3. Alcohol Contents

Alcohol content in HL and TL was measured using an alcoholmeter with temperature correction. Samples were transferred into a 100 mL graduated cylinder, the alcoholmeter and thermometer were inserted, equilibrated for 5 min, and readings were recorded.

#### 2.2.4. Total Acids

For FG and DG, the filtrates prepared in Section 2.2.2 were used for the total acid determination. A 50 mL filtrate, which is equivalent to 5 g of FG or DG, was titrated with 0.1 M NaOH to pH 8.2 using a PHS-3E pH meter (Shanghai Yidian Scientific Instrument Co., Ltd., Shanghai, China). The result was calculated according to Formula (3):

(3) $$\mathrm{Total}\;\mathrm{acids}\;(g/100\;g)\;=\;\frac{c\;\times \;V\;\times \;60\;\times \;100}{5}\;\times \;10^{-3}.$$

For HL and TL, 10 mL of sample was diluted with 20 mL distilled water, and titrated with 0.1 M NaOH to pH 8.2. The result was calculated according to Formula (4):

(4) $$\mathrm{Total}\;\mathrm{acids}\;(g/100\;\mathrm{mL})\;=\;\frac{c\;\times \;V\;\times \;60\;\times \;100}{10}\;\times \;10^{-3}.$$

where *c* is concentration of NaOH standard solution (mol/L); *V* is the titrant volume (mL); and 60 is the molar mass of acetic acid (g/mol).

#### 2.2.5. Total Esters

After total acid titration, 25 mL of 0.1 M NaOH solution was added to the flask, mixed, and refluxed for 30 min in boiling water. After cooling, the solution was titrated with 0.1 M H_2_SO_4_ until pH 8.7. Meanwhile, a blank titration using distilled water was conducted. The total esters were calculated:

For FG and DG, was calculated by Formula (5):

(5) $$\mathrm{Total}\;\mathrm{esters}\;(g/100\;g)\;=\;\frac{c_{1}\;\times \;(V_{b}\;-\;V_{a})\;\times \;88\;\times \;100}{5}\;\times \;10^{-3}$$

For HL and TL, was calculated by Formula (6):

(6) $$\mathrm{Total}\;\mathrm{esters}(g/100\;\mathrm{mL})\;=\;\frac{c_{1}\;\times \;(V_{b}\;-\;V_{a})\;\times \;88\;\times \;100}{10}\;\times \;10^{-3}$$

where *c*_1_ is the concentration of H_2_SO_4_ standard solution (mol/L); *V*_a_ and *V*_b_ are the titrant volumes for sample and blank (mL); and 88 is the molar mass of ethyl acetate (g/mol).

### 2.3. Analysis of VCs

#### 2.3.1. SPME Extraction

Samples were placed in 20 mL headspace vials under the optimal extraction conditions determined by preliminary single-factor experiments (Table 1), with saturated NaCl solution or solid NaCl added. Menthol (Shanghai Aladdin Biochemical Technology Co., Ltd., Shanghai, China) was used as the internal standard for all samples [23]. It was added at 0.835 µg/mL for FG and DG, and at 200 µg/mL for HL and TL. After the vials were sealed, they were put into a magnetic stirring water bath (Shanghai Lichen Bangxi Instrument Technology Co., Ltd., Shanghai, China) to equilibrate for 10 min. Then a 50/30 µm DVB/CAR/PDMS SPME fiber (Supelco, Bellefonte, PA, USA) preconditioned at 250 °C for 20 min, was used to extract the VCs. Following extraction, the VCs on the fiber were desorbed in the GC-MS injection port at 230 °C for 5 min.

#### 2.3.2. GC-MS Analysis

VCs were analyzed using an Agilent 7890B GC coupled to a 5977B MSD (Agilent Technologies, Santa Clara, CA, USA) with a DB-WAX capillary column (30 m × 250 µm, 0.25 µm). The oven temperature program was as follows: initial temperature of 40 °C was maintained for 2 min, followed by an increase to 230 °C at a rate of 5 °C/min, with a final hold for 5 min. The injector and detector temperatures were both maintained at 250 °C. High-purity helium (He) was used as the carrier gas at a constant flow rate of 2.0 mL/min. Samples were analyzed in splitless mode. Mass spectrometric detection was performed in EI mode (70 eV), while ion source, quadrupole and transfer line temperatures were 230 °C, 150 °C and 280 °C, respectively. Full scan spectra were recorded from *m*/*z* 33 to 500. Compounds were identified by matching the mass spectra of each component with the NIST 17 mass spectra database. At last, their relative contents were calculated by dividing the isolated compound’s peak area by the internal standard’s peak area [24].

### 2.4. Statistical Analysis

Orthogonal partial least squares discriminate analysis (OPLS-DA) is a supervised multivariate statistical method widely applied in flavoromics [25]. OPLS-DA and variable importance in projection (VIP) were conducted using SIMCA 14.1 (Umetrics, Umeå, Sweden). Peak areas of VCs were imported into SIMCA 14.1 (Umetrics, Umeå, Sweden) and normalized using UV scaling. The model robustness is further assessed by 200 random permutation tests. Model performance was evaluated using *R*^2^ (goodness-of-fit) and *Q*^2^ (predictive ability), with *Q*^2^ derived from 7-fold cross-validation. Typically, both *R*^2^ and *Q*^2^ values are required to be greater than 0.5, with values closer to 1 indicating better performance [26]. VCs were performed using SIMCA 14.1 (Umetrics, Umeå, Sweden) to calculate VIP values. Normality and homogeneity of variance were examined for the VCs data. As both assumptions were violated, non-parametric Kruskal-Wallis tests were subsequently performed in SPSS 25 (IBM, Armonk, NY, USA) on VCs with VIP > 1 [27]. VCs simultaneously meeting both criteria (VIP > 1 and *p* < 0.05) were considered significantly different.

Line plots and stacked bar charts were generated with Origin 2022 (OriginLab Corporation, Northampton, MA, USA). Heatmaps and Upset plots were constructed using TBtools v2.1 (South China Agricultural University, Guangzhou, China). Redundancy analysis (RDA) was performed in R 4.3.3 (the R Foundation for Statistical Computing, Vienna, Austria).

## 3. Results and Discussion

### 3.1. Physicochemical Indicators

**Figure 2:**
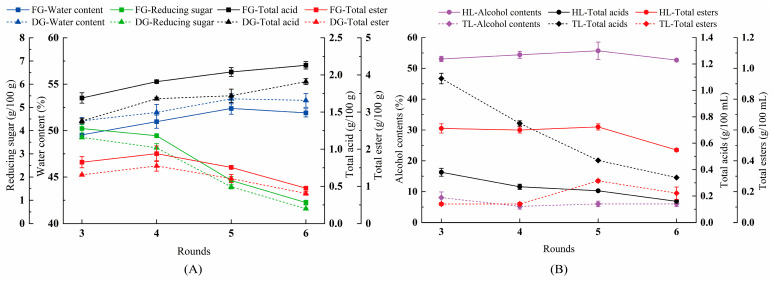
Changes of physicochemical indicators from the 3rd to the 6th rounds. (**A**) Water contents, reducing sugars, total acids, and total esters in fermented grains (FG) and distilled fermented grains (DG). (**B**) Alcohol contents, total acids, and total esters in heart liquor (HL) and tail liquor (TL). Colors represent different parameters: blue for water content, green for reducing sugars, black for total acids, red for total esters, and purple for alcohol content.

From the 3rd to the 6th round, the water contents of FG and DG first increased and then declined (Figure 2A), varying from 49.55% to 52.39% in FG and 51.05% to 53.43% in DG. Maintaining an appropriate water content is crucial for supporting the fermentation process, as excessive moisture may inhibit the growth of anaerobic microorganisms, while insufficient moisture may prevent adequate hydration of the grains [28,29]. In contrast, the reducing sugar contents decreased sharply across the rounds, falling from 4.09 to 0.90 g/100 g in FG and from 3.71 to 0.64 g/100 g in DG, with the most pronounced reduction occurring from the 4th to 5th round. Within the same round, DG consistently contained lower reducing sugar levels than FG. Throughout fermentation, microorganisms continuously consume reducing sugars to sustain their growth, which gradually lowers the sugar content in FG as the process advances [29]. In the subsequent distillation stage, sugars in the complex matrix may participate in Maillard reactions, undergo caramelization, or thermally decompose [30], further reducing the reducing sugar levels in DG. As for the alcohol content, it remained relatively stable, ranging from 52.67% to 55.70% in HL and from 5.23% to 8.07% in TL (Figure 2B).

Total acid contents in FG and DG increased with successive rounds, from 1.69 to 2.13 g/100 g in FG and from 1.38 to 1.91 g/100 g in DG (Figure 2A). In contrast, the total acid contents decreased in HL and TL from the 3rd to the 6th rounds (Figure 2B). The increased acid content in FG primarily originated from the activity of acid producing bacteria [9]. Within the same round, DG always contained less acid than FG, reflecting the migration of volatile acids in FG into HL and TL, while TL invariably exhibited higher acid content than HL. The elevated acid in TL is likely due to the accumulation of high-boiling-point acids in later distillation fractions [31]. For example, the high-boiling-point octanoic acid in TL was consistently higher than that in HL within the same round (Table S1) [3,6,9,32,33,34,35,36,37,38,39,40].

The total ester contents in FG, DG, HL and TL exhibited a similar trend, rising initially and then declining from the 3rd to the 6th rounds. Within the same round, FG always contained more esters than DG, while HL exhibited higher ester contents than TL. The higher ester content in FG compared with DG can be attributed to the transfer of partial esters into liquors during distillation, whereas the higher ester content in HL compared to TL is mainly due to the preferential migration of low-boiling-point esters into earlier distillation fraction (HL). For example, ethyl hexanoate, which has a lower boiling point, was present in higher amounts in HL than that in TL within each round (Table S1).

During the fermentation of sauce-aroma *Baijiu*, microorganisms consume reducing sugars from cereal materials while substantial amounts of organic acids are produced [11,41]. These acids not only act as important flavor compounds in *Baijiu*, but also participate in esterification reaction, which facilitates ester VCs formation [27,42]. Therefore, the total acid contents in FG and DG increased substantially while the total ester contents reached to it maximum in HL5 and TL5 (Figure 2).

### 3.2. Profiling and Quantification of VCs

**Figure 3:**
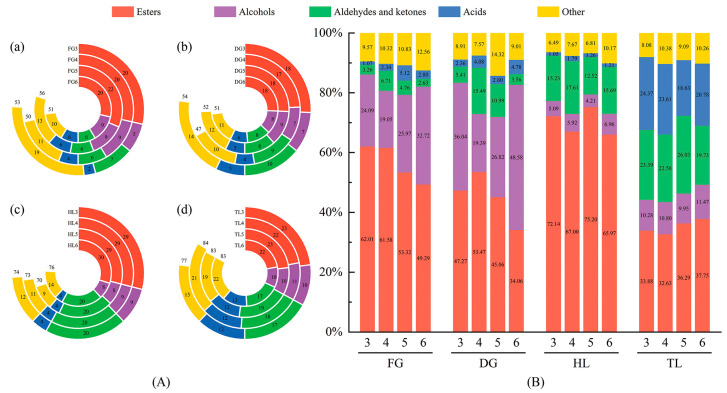
Quantity (**A**) and relative content proportion (**B**) of volatile compound categories. In panel (**A**), (**a**) fermented grains (FG), (**b**) distilled fermented grains (DG), (**c**) heart liquor (HL) and (**d**) tail liquor (TL) represent the number of compounds in each group. Colors indicate compound categories: red for esters, purple for alcohols, green for aldehydes and ketones, blue for acids, and yellow for other.

A total of 76, 73, 80 and 93 VCs were identified in FG, DG, HL and TL, respectively (Table S1). Esters, alcohols, aldehydes and ketones, and acids were the dominant VC classes and their content proportional distributions were shown in Figure 3. Esters were consistently the most diverse class of VCs across all rounds and sample types. In FG and DG, no obvious differences were observed in the numbers of alcohol VCs and aldehyde and ketone VCs (Figure 3A). In contrast, in HL and TL, aldehyde and ketone VCs outnumbered alcohol VCs. Notably, TL contained substantially more acid VCs than FG, DG, or HL (Figure 3A,B).

In addition to having the highest diversity, ester VCs were also the most abundant VCs, accounting for 49.29–62.01% of total VCs in FG, 34.06–53.47% in DG, 65.97–75.20% in HL, and 32.63–37.75% in TL, respectively (Figure 3B). In FG and DG, ester contents gradually decreased with successive rounds, whereas they peaked in HL5 and TL6. In FG and DG, the major ester components were ethyl lactate, ethyl phenylacetate, and ethyl hexadecanoate, which primarily originate from microbial metabolism [43]. On the other hand, the dominant esters in HL and TL included ethyl hexanoate, ethyl pentanoate, ethyl phenylacetate, and ethyl caprylate (Table S1). And these dominant compounds are recognized as key aroma-active esters contributing to the characteristic flavor of sauce-aroma *Baijiu* [5,44]. Notably, several medium-chain fatty acid ethyl esters, such as ethyl decanoate and dodecanoic acid ethyl ester, were abundant in FG and DG but not detected in HL and TL (Table S1). Due to their high boiling points [45], these esters are difficult to distill and therefore cannot be efficiently transferred into the liquor. In addition, the high temperature during distillation can promote thermal degradation or transformation reactions, resulting in a decrease content of ester from FG to DG in the same round. Compared with FG and DG, more unique esters appeared in HL and TL. For example, ethyl hexanoate and ethyl pentanoate, both are important flavor compounds in sauce-aroma *Baijiu*, were consistently detected in all HL and TL rounds but were completely absent in FG and DG (Table S1). These newly formed esters after distillation may arise from chemical reactions occurring within the complex matrix of FG. In the presence of acids, alcohols, and high-temperature catalytic conditions, extensive esterification reactions can generate large amounts of esters [27], which play crucial roles in shaping the characteristic aroma of sauce-aroma *Baijiu*.

Alcohol VCs are another flavor category, serving as auxiliary components that modulate the aroma and flavor of *Baijiu* [46]. The alcohol VCs abundant in FG and DG but less in HL and TL (Figure 3B). Among them, 3-methyl-1-butanol was most abundant in HL, whereas phenylethyl alcohol predominated in FG, DG, and TL (Table S1). 3-methyl-1-butanol is a key characteristic marker of sauce aroma *Baijiu* [9]. In our study, its content increased from HL3 to HL6, consistent with the typical profile of southern sauce-aroma *Baijiu* [9]. In addition, the phenylethyl alcohol, 3-octanol, 2-nonanol and 2-methyl-1-propanol are also important contributors to the mellow texture and enhanced aftertaste of *Baijiu* [47,48], which also shared the same increasing pattern as 3-methyl-1-butanol (Table S1). In contrast, propylene glycol and 2,3-butanediol were abundant in grains but disappeared in liquors. However, their strong hydrophilicity prevents their mass transfer from being enhanced by the ethanol “solvent effect” [49], causing them to remain largely in DG after the distillation process (Table S1).

Different from alcohol VCs, the aldehyde and ketone VCs were abundant in HL and TL but scarce in FG and DG (Figure 3B). Generally, aldehyde and ketone VCs are important in enhancing the fragrance and mouthfeel and contribute to the distinctive flavor of sauce aroma *Baijiu* [26]. Furfural, detected in DG, HL and TL, but absent in FG, which reached 6586.88 ± 342.17 µg/L in HL5 but decreased to 5632.7 ± 506.11 µg/L in HL6 (Table S1). Furfural, like 3-methyl-1-butanol, is a characteristic flavor compound in southern sauce-aroma *Baijiu* and is strongly associated with roasted aromas [9]. While high-quality *Baijiu* often contains relatively high amounts of furfural, an overabundance can introduce undesirable burnt notes and lead to an unpleasant drinking experience [24]. Apart from furfural, benzaldehyde and benzeneacetaldehyde are two other aldehydes strongly associated with base liquor quality [24], and their contents increased progressively across the fermentation rounds (Table S1). Benzaldehyde is mainly responsible for the characteristic “sauce” aroma [50], while benzeneacetaldehyde contributes floral notes that enhances the distinctive flavor profile of sauce-aroma *Baijiu* [33].

Acid VCs were least abundant in FG, DG, and HL but accounted for up to 24.37% of total VCs in TL. As illustrated in Figure 3, acid VCs accounted for only 1.07–5.12% of total VCs in FG, 2.36–4.78% in DG, and 1.21–1.79% in HL, whereas they contributed 18.63–24.37% of total VCs in TL. In addition to participating in esterification reactions with alcohols, these accumulated acids in the liquid fraction also help prolong the aftertaste of sauce-aroma *Baijiu* [14]. Acetic acid was the most abundant acid in HL, followed by hexanoic acid (Table S1). Besides contributing to *Baijiu* aroma, acetic acid also serves as a key precursor for the formation of acetate esters and plays an important role in regulating the microbial community during fermentation [15,51]. Hexanoic acid, characterized by sour, sweaty, and cheesy notes, is considered an important aroma marker of sauce-aroma *Baijiu*. Compared with FG and DG, many acid VCs were present only in HL and TL (Table S1), which may be likely formed or released through chemical reactions occurring during distillation. Furthermore, the greater diversity of acids in TL relative to HL can be attributed to differences in their physicochemical properties. The acids with lower boiling points and higher ethanol solubility tend to accumulate in HL, where the alcohol content is higher, whereas higher-boiling and higher water-soluble acids are more likely to concentrate in TL [52]. Although the specific acids in TL have not been identified as key aroma contributors in *Baijiu*, some of them, such as heptanoic acid, which are known to be important volatile components in other fermented products, such as vinegar [53].

Furthermore, other classes of VCs were also detected in the tested samples (Table S1), contributing to the enrichment of the flavor of sauce-aroma *Baijiu*. For instance, 2,3,5-trimethylpyrazine with sweet, nutty and smoky notes [39], 2,4-di-tert-butylphenol with phenol note [3,54], and 2-acetyl-5-methylfuran contributed to green and roast notes [32], were also found in HL and TL.

Considering all VCs, esters were the dominant aroma constituents in *Baijiu*, and their diversity and abundance largely determined the quality of the final *Baijiu* product [5,27]. Consistent with previous studies [55], the HL5 in our work also exhibited the highest ester contents. In addition, a balanced composition of alcohols, aldehydes and ketones, acids, and other minor compounds is indispensable for *Baijiu* quality. The insufficient levels of them lead to weak aroma, whereas excessive levels may cause undesirable off-odors and unhealthy effect [56,57]. Therefore, when considering the overall VC profile, the precise proportions among different classes of compounds make HL4, HL5, and HL6 generally recognized as the best base liquors for producing the final product [58]. On the other hand, the particularly high levels of acids in TL, together with its unique VC composition, indicate that TL could be further utilized for producing fermented by-products such as vinegar [13]. Moreover, the grains remaining after brewing contain not only oligopeptides but also characteristic aroma compounds. Thus, they have the potential to be developed into value-added products rather than being used solely as livestock feed [59].

### 3.3. Screening of Key Differential VCs

**Figure 4:**
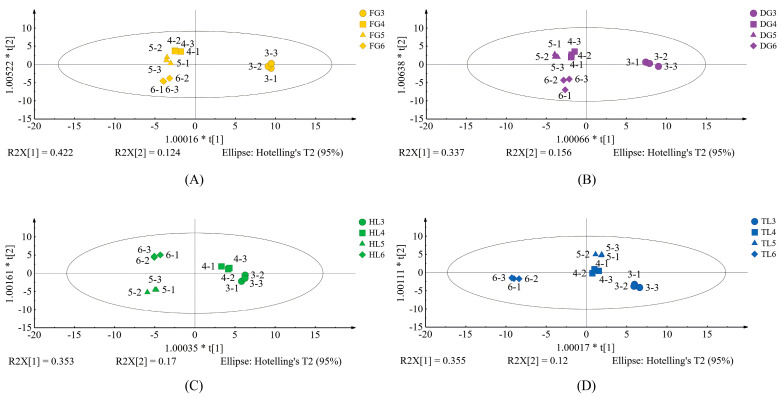
Orthogonal partial least squares discriminate analysis (OPLS-DA) score plots of fermented grains (FG), distilled fermented grains (DG), heart liquor (HL) and tail liquor (TL) from the 3rd to 6th rounds. (**A**) FG, (**B**) DG, (**C**) HL, and (**D**) TL. Each symbol represents a sample from a specific round, with 95% confidence ellipses shown. Clear separation among rounds indicates significant differences in volatile compounds.

OPLS-DA revealed clear inter-round differentiation among FG, DG, HL, and TL samples, with all models showing good performance (*R*^2^ and *Q*^2^ > 0.5) and no evidence of overfitting (Figure S1; Table S2) [56]. The results also revealed that the VCs profiles of FG and DG exhibited the same trend, in which VCs profiles from the 3rd round showed the greatest divergence, whereas those from the 4th to 6th rounds were similar (Figure 4). In addition, VCs from HL and TL exhibited comparable variation patterns, with being the most distinct in both at the 6th round. However, the VCs of TL3, TL4 and TL5 clustered together, whereas the VCs in HL3 and HL4 were similar but diverged markedly in HL5 (Figure 4).

**Figure 5:**
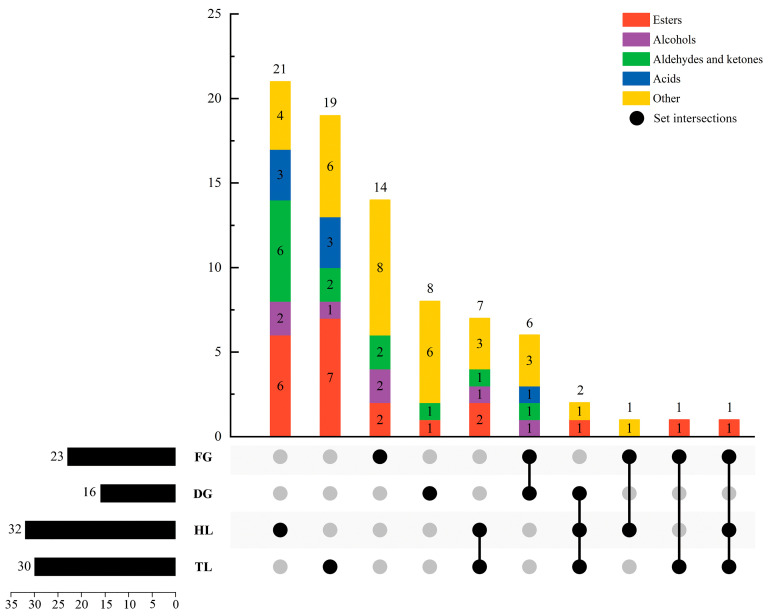
Upset plot of key differential volatile compounds (VCs) of fermented grains (FG), distilled fermented grains (DG), heart liquor (HL) and tail liquor (TL). Upset plot showing the distribution of key differential VCs among sample types. Black dots indicate set intersections, the black bar at the lower left gives the total number of key differential VCs for samples, and the colored bars show the number of compounds shared across intersections: red for esters, purple for alcohols, green for aldehydes and ketones, blue for acids, and yellow for other.

VCs with VIP > 1 were tested by the Kruskal-Wallis test (*p* < 0.05). Consequentially a total of 23, 16, 32 and 30 key differential VCs were identified in FG, DG, HL and TL, respectively (Figure 5). Among them, HL contained the largest number of unique key differential VCs, followed by TL, FG, and DG, with **21**, **19**, **14**, and **8** compounds, respectively. In terms of shared VCs, **10** were common to HL and TL, **6** to FG and DG, **2** to DG, HL, and TL, **2** to FG and HL, **2** to FG and TL, and **1** to FG, HL, and TL, as shown in Figure 5 and listed in Table S3.

**Figure 6:**
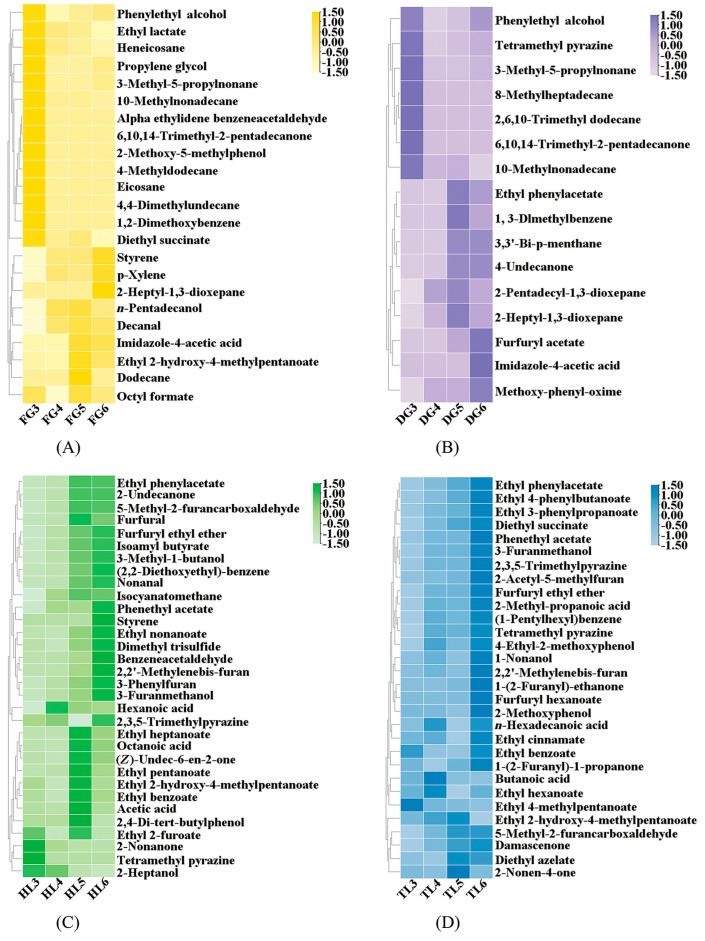
Heatmaps of key differential VCs of fermented grains (FG), distilled fermented grains (DG), heart liquor (HL) and tail liquor (TL). (**A**) FG, (**B**) DG, (**C**) HL, and (**D**) TL. Color gradients from light to dark represent relative abundance from low to high based on row-normalized data; vertical comparisons are not applicable.

The key differential VCs in all samples were normalized and visualized in a clustered heatmap (Figure 6). As shown in Figure 6A, most key differential VCs from in FG were concentrated in FG3, with phenylethyl alcohol and ethyl lactate exhibiting the strongest signal intensity. In DG, the key differential VCs were primarily concentrated in DG3 and DG5, among which phenylethyl alcohol and ethyl phenylacetate showed the highest abundance (Figure 6B). Consistently, Zeng et al. [19] also identified phenylethyl alcohol, ethyl lactate, and ethyl phenylacetate as key differential VCs in sauce-aroma *Baijiu* across different rounds. In addition, relatively high levels of diethyl succinate and octyl formate impart fruity notes to FG [33], while tetramethyl pyrazine contributes nutty and roasted notes to DG [40], collectively enhancing the overall flavor profile of the *Baijiu*.

For HL, the differential VCs were mainly concentrated in HL5 and HL6, whereas for TL they were concentrated in TL6 (Figure 6C,D). In HL, ethyl phenylacetate and furfural were abundant but displaying a rise-fall trend from the 3rd to 6th rounds. Ethyl phenylacetate, a key aroma component in sauce-aroma *Baijiu* [44], peaked in HL5 and then declined slightly, consistent with the findings of Wu et al. [9]. This compound not only differentiates distillation rounds but also imparts characteristic nutty and bean-like notes to HL. Meanwhile, esters such as ethyl heptanoate, ethyl pentanoate, and ethyl benzoate also peaked in HL5, contributing predominantly floral and fruity aromas to HL. Furfural, another important VC in HL, is mainly derived from the decomposition of pentoses in auxiliary materials such as rice husks during fermentation [60] and contributes almond-like notes [34]. In addition, hexanoic acid, 3-furanmethanol, 2,3,5-trimethylpyrazine and tetramethyl pyrazine are also key contributors to the aroma of sauce-aroma *Baijiu* [61]. Collectively, the pronounced enrichment of these differential aroma compounds suggests that HL from these rounds exhibits a more abundant aroma-active compound profile, making it more suitable for use in the production of the final *Baijiu* product.

Although TL contains several odor-active VCs such as 1-(2-furanyl)-ethanone and 1-(2-furanyl)-1-propanone [62], it also has a high alcohol contents, abundant organic acids, and other pleasant VCs with floral, fruity, and creamy notes, including ethyl phenylacetate, diethyl succinate, and acetoin [9,34], making it a promising substrate to produce other fermented products [13]. Moreover, as the rounds progressed, the contents of 2-acetyl-5-methylfuran, 3-furanmethanol, 2,3,5-trimethylpyrazine and tetramethyl pyrazine gradually increased, all of which contribute to the characteristic aroma of sauce-aroma *Baijiu* [61]. Notably, TL6 exhibited the highest total concentration of flavor compounds and the greatest proportion of esters (Figure 3B and Figure 6D), highlighting its considerable potential for further utilization.

### 3.4. Correlation Analysis Between Physicochemical Indicators and VCs

**Figure 7:**
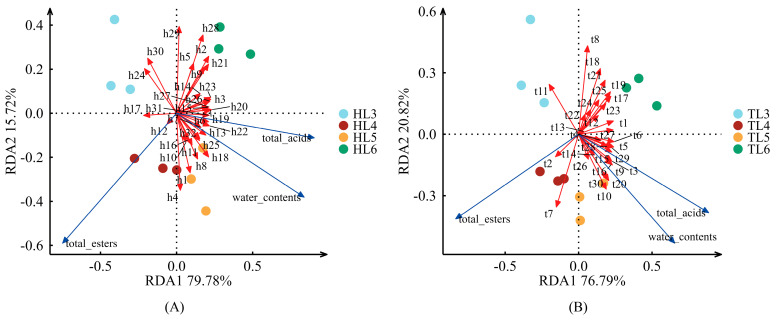
Redundancy analysis (RDA) between physicochemical indicators of fermented grains (FG) and the key differential volatile compounds (VCs) in (**A**) heart liquor (HL) and (**B**) tail liquor (TL). Physicochemical indicators and VCs are represented by blue and red lines with arrow. Compounds **h1**–**h32** denote the key differential VCs of HL, and **t1**–**t30** denote those of TL. Colored dots represent the samples from different rounds: blue for the 3rd round, red for the 4th round, yellow for the 5th round, green for the 6th round.

To further explore the relationships between the physicochemical indicators of FG and the VCs of liquors, RDA was performed. After screening, water contents, total acids, and total esters were retained for subsequent analysis [42]. Figure 7 showed the relationship between the three physicochemical indicators of FG and the key differential VCs of HL and TL, in which arrow length represents the magnitude of the correlation, while the angle between arrows indicates the direction of the relationship [42].

The RDA results showed that the two axes explained 95.5% (Figure 7A) and 97.61% (Figure 7B) of the total variance in key differential VCs, respectively, indicating strong associations between physicochemical indicators and VC profiles. Based on the arrow lengths, most VCs in HL and TL were associated with the 5th and 6th rounds, suggesting that their concentrations increased in later stages. The angles between vectors indicated that total acids and water contents were positively correlated with ester VCs such as **h6**, **h26** and **t4**, alcohol VCs such as **h20**, aldehyde and ketone VCs such as **h13**, **h23** and **h31**, as well as other compounds including **t15**, **t29** and **t30**. In addition, total esters were positively correlated with VCs such as **h12**, **h17**, **t2**, and **t7** (Figure 7 and Table S4). The acute angles formed between the vectors of water contents and most VCs, as well as between total acids and most VCs, indicating that these two parameters from FG were positively correlated with the majority of VCs and were key physicochemical indicators linked to the flavor profile of HL and TL.

The essence of *Baijiu* brewing is essentially the process of microbial growth and the accumulation of their metabolic products [1], and the growth and metabolic activities of these microorganisms are strongly governed by the physicochemical properties of the fermentation environment [46,63]. During solid-state fermentation, physicochemical properties such as starch content, moisture, acidity, and reducing sugars change continuously as fermentation progresses; these changes affect microbial metabolic processes and consequently influence the formation of flavor compounds [9]. During stacking fermentation, reducing sugars serve as the key driver of microbial succession; during pit fermentation, acidity and related parameters act as critical regulatory factors. Previous studies identified total acids and water contents as the primary environmental factors driving the evolution of bacterial and fungal communities [46,64]. As fermentation progresses, both water and acid contents generally increase throughout the entire process [22,65]. Changes in water content not only affect the levels of VCs but also influence the overall flavor profile and structural characteristics of the base liquor [66]. Moreover, increasing acidity favors the dominance of acid-tolerant microorganisms, leading to marked shifts in microbial community composition [65,66]. For instance, acid-tolerant microorganisms such as *Lactobacillus* spp. multiply rapidly and become the prevailing population [63], which actively drive fermentation and generate organic acid precursors for ester synthesis. Huang et al. [67] found that *Lactobacillus* spp. is significantly positively correlated with esters including ethyl phenylacetate and plays an important role in ester synthesis during the fermentation of sauce-aroma *Baijiu*. *Aspergillus*, the core fungal genus in sauce-aroma *Baijiu* fermentation, is central to saccharification, fermentation and esterification [68] and shows positive correlations with both water content and acidity [19,46]. This genus produces proteolytic enzymes and various lyases that not only facilitate starch saccharification and protein hydrolysis but also promote the formation of VCs such as 3-methyl-1-butanol [69].

In summary, strong correlations exist between physicochemical indicators of FG and key VCs in HL and TL. Accordingly, precise control of these key parameters during fermentation is expected to contribute to improvements in liquor aroma complexity and overall quality.

## 4. Conclusions

The dynamic changes of physicochemical indicators and VCs profiles were systematically investigated and compared from FG, DG, HL and TL of the 3rd to 6th rounds in the brewing process of sauce-aroma *Baijiu*. The results demonstrate that the physicochemical indicators of both FG and DG exhibited consistent dynamic trends across the rounds and the indicators of FG were strongly associated with key differential VCs in HL and TL. Notably, HL5 contained the highest total VCs and the greatest proportion of pleasant esters, suggesting that it may represent the most favorable raw liquor for final *Baijiu* production, whereas the by-product TL6 with high acid content and the greatest proportion of esters appeared most suitable for further applications such as vinegar production.

Overall, this study elucidates the interrelationships among FG, DG, HL and TL, as well as the correlation between the physicochemical indicators of the FG and the resulting liquor, thereby providing a theoretical basis for *Baijiu* quality control, evaluating HL quality across different rounds, and promoting the efficient utilization of TL. However, it should be noted that although aroma-related compounds were extensively analyzed in this study, sensory evaluation was not performed, and thus the direct relationship between the identified VCs and human sensory perception remains to be further validated. Additionally, as this study focused on sauce-aroma *Baijiu*, the generalizability of the findings to other *Baijiu* varieties is limited. Future research may integrate sensory analysis and omics methods to validate the relationship between VCs and human sensory perception, uncover the formation pathways of key VCs and the interplay between microbial communities and VCs, and to validate these findings across multiple *Baijiu* types.

## Figures and Tables

**Table 1:** Headspace solid-phase microextraction conditions (HS-SPME) for volatile compounds from fermented grains (FG), distilled fermented grains (DG), heart liquor (HL) and tail liquor (TL).

Samples	Amount	NaCl Solution/Solid NaCl	Internal Standard (µL)	HS-SPME Conditions		
				Equilibrium Time (min)	Extraction Temperature (°C)	Extraction Time (min)
FG	1.5 g	5 mL	50	10	50	30
DG	1.5 g	5 mL	50	10	50	30
HL	8 mL	2.5 g	25	10	30	50
TL	8 mL	3.0 g	25	10	40	50

## Data Availability

The original contributions presented in this study are included in the article/Supplementary Material. Further inquiries can be directed to the corresponding authors.

## Supplementary Materials

The following supporting information can be downloaded at: https://www.mdpi.com/article/10.3390/foods15122211/s1, Table S1: VCs contents of FG, DG, HL and TL; Table S2: Performance statistics of the OPLS-DA models; Table S3: Upset-plot dataset of unique and shared key differential VCs; Table S4: Key differential VCs used in RDA; Figure S1: OPLS-DA permutation test.

## Abbreviations

The following abbreviations are used in this manuscript:

FG	Fermented grains
DG	Distilled fermented grains
HL	Heart liquor
TL	Tail liquor
HdL	Head liquor
VCs	Volatile compounds
OPLS-DA	Orthogonal partial least squares discriminate analysis
HS-SPME/GC-MS	Headspace solid-phase microextraction coupled with gas chromatography–mass spectrometry
VIP	Variable importance in projection
RDA	Redundancy analysis
