# Supplementary material for: Changes of Physicochemical Indicators and Volatile Compounds in Grains and Liquors During the Sauce-Aroma Baijiu Brewing Process
Source: Foods. 2026 Jun 19;15(12):2211. doi: 10.3390/foods15122211 (PMC13298116; doi:10.3390/foods15122211)
Supplement: Supplementary file 1 [file foods-15-02211-s001.zip › Supplementary captions.pdf]

Appendix of supplementary data

Table S1. Volatile compounds (VCs) contents of fermented grains (FG), distilled fermented grains (DG), heart liquor (HL) and tail liquor (TL). "-" indicates that the component was not detected. "X" indicates that the odor characteristic has not been reported. In the "Odor characteristics" column, blue text indicates positive valence associated with preference, whereas red text indicates negative valence associated with rejection.

Table S2. Performance statistics of the orthogonal partial least squares discriminate analysis (OPLS-DA) models.

Table S3. Upset-plot dataset of unique and shared key differential volatile compounds (VCs).

Table S4. Key differential volatile compounds (VCs) used in redundancy analysis (RDA). Compounds h1-h32 are the key differential VCs of heart liquor (HL), and t1-t30 are those of tail liquor (TL).

Figure S1. Orthogonal partial least squares discriminate analysis (OPLS-DA) permutation test. (A) fermented grains (FG), (B) distilled fermented grains (DG), (C) heart liquor (HL), and (D) tail liquor (TL). The negative Y-intercept of the Q^2 regression line indicates that the OPLS-DA model was not overfitted.
