# Supplementary figures and images for: Changes of Physicochemical Indicators and Volatile Compounds in Grains and Liquors During the Sauce-Aroma Baijiu Brewing Process
Source: Foods. 2026 Jun 19;15(12):2211. doi: 10.3390/foods15122211 (PMC13298116; doi:10.3390/foods15122211)

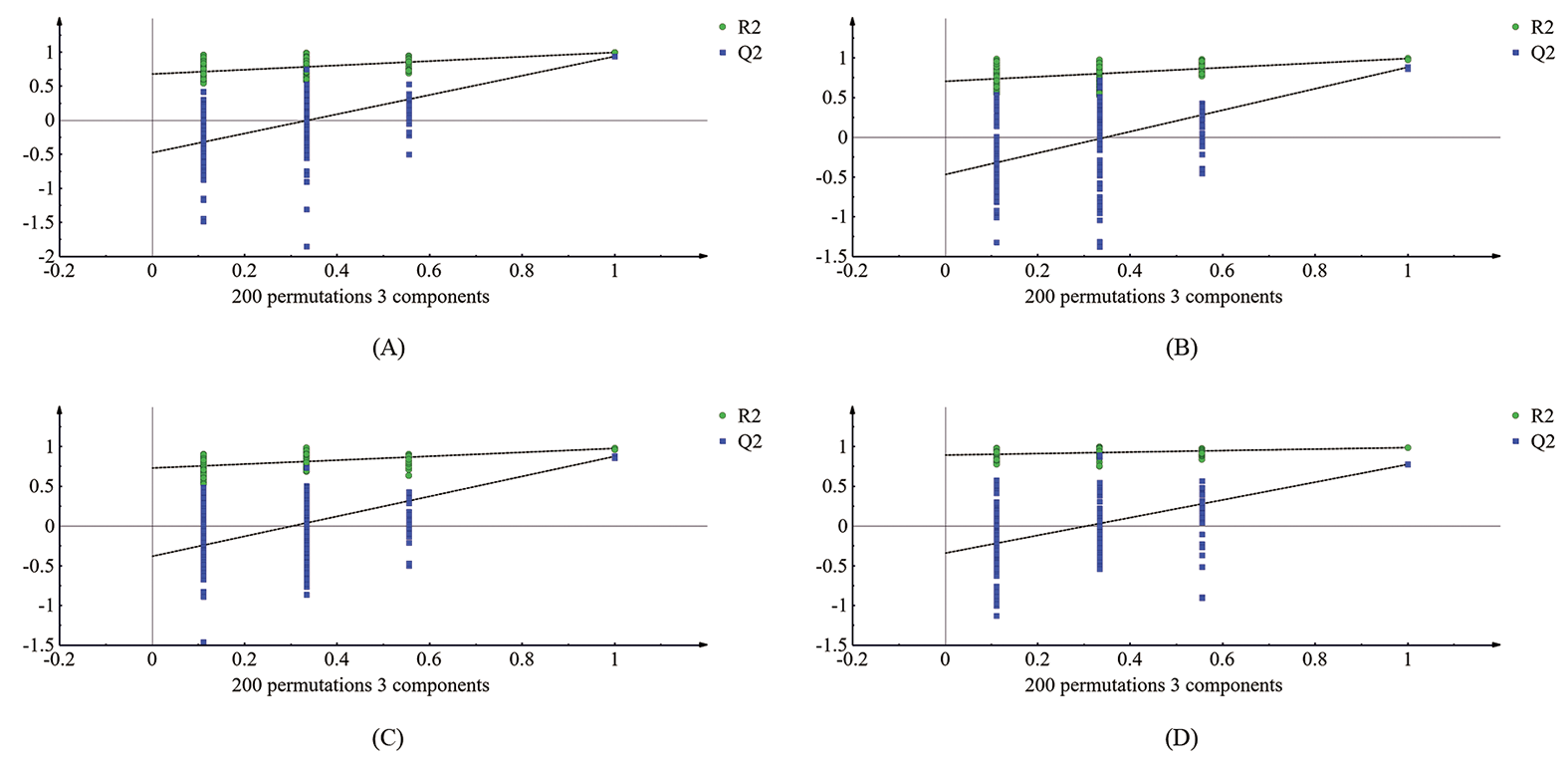

Supplement: Supplementary file 1 [file foods-15-02211-s001.zip › Figure S1.tif]
